# Identification of new immune subtypes of renal injury associated with anti-neutrophil cytoplasmic antibody–associated vasculitis based on integrated bioinformatics analysis

**DOI:** 10.3389/fgene.2023.1119017

**Published:** 2023-04-05

**Authors:** Lizhen Lin, Keng Ye, Fengbin Chen, Jingzhi Xie, Zhimin Chen, Yanfang Xu

**Affiliations:** ^1^ Department of Traditional Chinese Medicine, The First Affiliated Hospital, Fujian Medical University, Fuzhou, China; ^2^ Blood Purification Research Center, Department of Nephrology, The First Affiliated Hospital, Fujian Medical University, Fuzhou, China; ^3^ Fujian Clinical Research Center for Metabolic Chronic Kidney Disease, The First Affiliated Hospital, Fujian Medical University, Fuzhou, China; ^4^ National Regional Medical Center, Department of Nephrology, Binhai Campus of the First Affiliated Hospital, Fujian Medical University, Fuzhou, China; ^5^ Central Laboratory, The First Affiliated Hospital, Fujian Medical University, Fuzhou, China

**Keywords:** ANCA-associated vasculitis, renal injury, bioinformatics, biomarkers, immune subtypes, WGCNA

## Abstract

**Background:** Anti-neutrophil cytoplasmic antibody (ANCA)-associated vasculitis (AAV) is a systemic autoimmune disease that may lead to end-stage renal disease. However, few specifific biomarkers are available for AAV-related renal injury. The aim of this study was to identify important biomarkers and explore new immune subtypes of AAV-related renal injury.

**Methods:** In this study, messenger RNA expression profiles for antibody-associated vasculitis and AAV-associated kidney injury were downloaded from the Gene Expression Omnibus database. Weighted gene co-expression network analysis (WGCNA) was performed to identify the most relevant module genes to AAV. Key module genes from WGCNA were then intersected with AAV- and nephropathy-related genes from the Genecards database to identify key genes for AAV-associated kidney injury. Subsequently, the expression of key genes was validated in independent datasets and the correlation of genes with clinical traits of kidney injury was verified by the Nephroseq database. Finally, non-negative matrix factorization (NMF) clustering was performed to identify the immune subtypes associated with the key genes.

**Results:** Eight co-key genes (*AGTR2*, *ANPTL2*, *BDKRB1*, *CSF2*, *FGA*, *IL1RAPL2*, *PCDH11Y*, and *PGR*) were identifified, and validated the expression levels independent datasets. Receiver operating characteristic curve analysis revealed that these eight genes have major diagnostic value as potential biomarkers of AAV-related renal injury. Through our comprehensive gene enrichment analyses, we found that they are associated with immune-related pathways. NMF clustering of key genes identified two and three immune-related molecular subtypes in the glomerular and tubular data, respectively. A correlation analysis with prognostic data from the Nephroseq database indicated that the expression of co-key genes was positively co-related with the glomerular filtration rate.

**Discussion:** Altogether, we identifified 8 valuable biomarkers that firmly correlate with the diagnosis and prognosis of AAV-related renal injury. These markers may help identify new immune subtypes for AAV-related renal injury.

## 1 Introduction

Antineutrophil cytoplasmic antibody (ANCA)-associated vasculitis (AAV) is a group of systemic autoimmune diseases typified by inflammation, the destruction of small- and medium-sized blood vessels, and the presence of circulating ANCAs. Primarily, AAV includes microscopic polyangiitis (MPA), granulomatosis with polyangiitis (GPA), and eosinophilic granulomatosis with polyangiitis. AAV usually causes damage to multiple organs at the same time, with the lungs and kidneys being most commonly affected and patients typically presenting with pulmonary hemorrhage and acute kidney injury. Patients with untreated AAV experience rapid progression of their disease, and the cause of death is primarily renal or respiratory failure (Cornec et al., 2016). The occurrence and progression of AAV correlate with multiple factors from the point of view of science and research, such as genetic susceptibility, cellular and environmental factors, and infection (Lee et al., 2012; Lyons et al., 2019). Genes and genetics are involved in the development of AAV autoimmunogenesis (Jennette et al., 2013; Jennette and Falk, 2014a; Kallenberg, 2014a; Jennette and Falk, 2014b). In recent years, immunosuppressive therapy has significantly improved the prognosis of patients with AAV (Kallenberg, 2014b), but early detection of AAV, evaluation of the treatment response, and outcome prediction are still considered challenging.

AAV-related renal injury mainly manifests as symptoms of hematuria, proteinuria, and abnormal renal function, and generally results in a rapid deterioration of renal function. Several studies have shown that severe AAV carries a high mortality rate, approaching 80% in untreated patients (Shi, 2017). The 5-year survival rates for patients with GPA and MPA, respectively, are 74%–91% and 45%–76% (Robson et al., 2015). A study of children with AAV in China found that AAV occurs most commonly in adolescent girls. Renal impairment was the most common symptom, with 58.8% of patients progressing to end-stage renal disease within a median period of 3 months (Wu et al., 2021a). It is clear that renal involvement is potentially life-threatening for patients, and exploring the molecular mechanisms underlying the onset and progression of AAV-related kidney injury and identifying new therapeutic targets could contribute to the early diagnosis and treatment of this disease and improve the prognosis.

More recently, high-throughput sequencing technologies have generated massive bioinformatics data, facilitating access to more accurate gene-expression profiles, the identification of disease-related genes and drug targets, and the analysis of the pathogenesis of complex diseases. Bioinformatics analysis has significant advantages for understanding the pathophysiological mechanisms of hereditary AAV. Numerous bioinformatics datasets in the Gene Expression Omnibus (GEO) database have been analyzed to uncover the pathogenesis of AAV. However, there are still relatively few bioinformatics studies of biomarkers and key pathways for AAV-related kidney injury.

In this study, we used bioinformatics approaches to screen for key genes participating in AAV-related kidney injury from multiple AAV dataset sources. In addition, using Gene Ontology (GO)/Kyoto Encyclopedia of Genes and Genomes (KEGG) and gene set enrichment analysis (GSEA) pathway analyses, the signaling pathways in which key genes were significantly enriched were clustered. Non-negative matrix factorization (NMF) and single-sample GSEA (ssGSEA) were performed to explore new subtypes of AAV-related kidney injury and immune infiltration. This study aimed to identify key genes and pathways to explore new subtypes of AAV-related kidney injury and provide potential candidate biomarkers for diagnosis, survival prediction, and drug targets for AAV-related kidney injury.

## 2 Materials and methods

### 2.1 Data download

**FIGURE 1:**
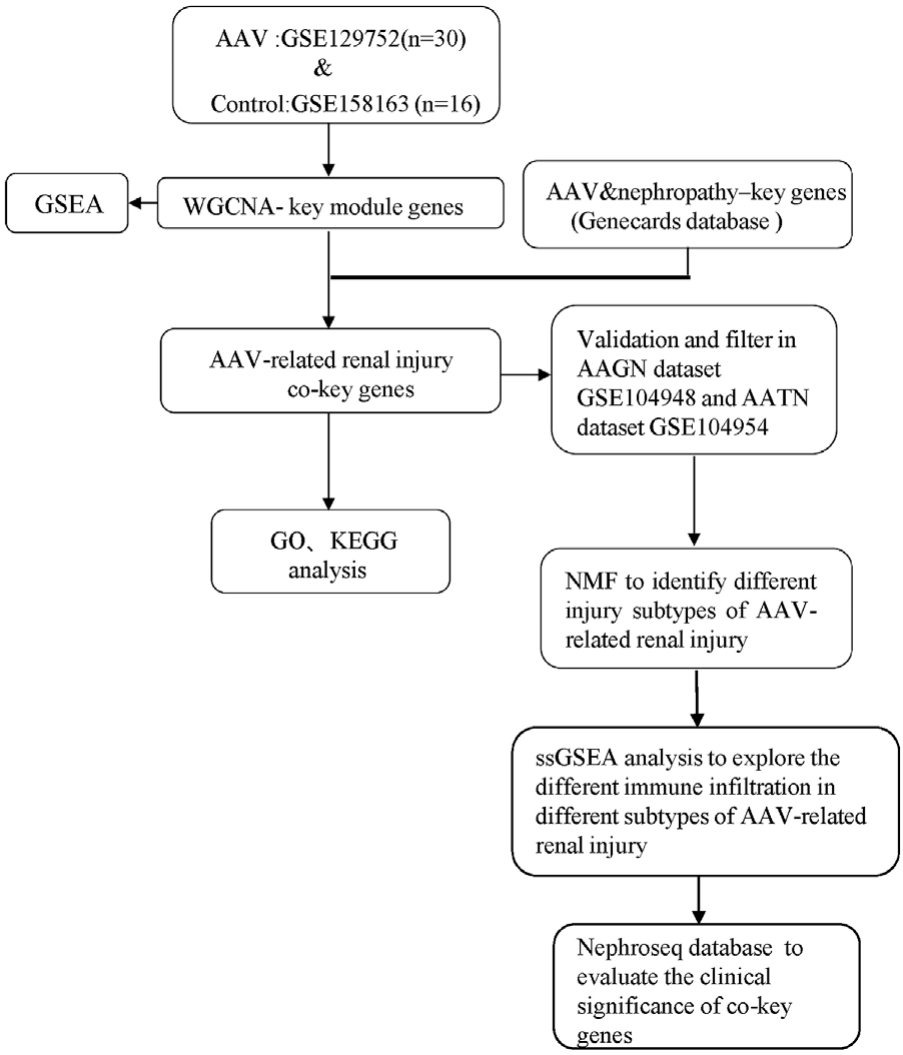
Study workflow. Anti-neutrophil cytoplasmic antibody–associated vasculitis (AAV), weighted gene co-expression network analysis (WGCNA), gene set enrichment analysis (GSEA), Gene Ontology (GO), Kyoto Encyclopedia of Genes and Genomes (KEGG), and non-negative matrix factorization (NMF).

We searched the GEO database (https://www.ncbi.nlm.nih.gov/geo/) (Barrett et al., 2013) for human AAV-, ANCA-associated glomerulonephritis (AAGN), and ANCA-associated tubulointerstitial nephritis (AATN)- related gene profiles using the keywords “ANCA,” “AAV,” “AAGN,” and “AATN.” Data from 30 AAV patients in the GSE129752 dataset (Gill et al., 2021) based on the GPL10999 platform (Illumina Genome Analyzer IIx, *Homo sapiens*) were used for subsequent analysis.The GSE158163 dataset (Grayson et al., 2018) is based on the GPL16791 platform (Illumina HiSeq 2500, *H. sapiens*), from which we extracted data from 16 control samples (virus negative and positive control groups) for subsequent analysis. The GSE104948 dataset is based on the GPL22945 platform (Affymetrix Human Genome U133 Plus 2.0 Array) and the GPL24120 platform (Affymetrix Human Genome U133A Array), and we extracted glomerular expression data from 21 normal and 22 AAGN patient samples. The GSE104954 dataset (Grayson et al., 2018) was also based on the GPL22945 platform and the GPL24120 platform, and we extracted renal tubular expression data from 21 control and 21 AATN patients (Figure 1).

### 2.2 Data pre-processing

For the RNA sequencing data, the sequencing count values of the GSE129752 and GSE158163 datasets were downloaded and converted to Fragments Per Kilobase of exon model per Million mapped fragments (FPKM) values using the biomaRt package in R. Since the optimal input matrix type for WGCNA was FPKM, the FPKM values were no longer converted to TPM values. Next, the FPKM matrices of the two datasets were subjected to missing value completion and data cleaning. We removed rows (genes) with NA values greater than 50% and columns (samples) with NA values greater than 50%, and furthermore, we used the impute.knn function of the R package impute to perform missing value completion by setting the Number of neighbors to K to complete the missing data. The expression values were taken as log2 (Exp+1) and the mean value of the duplicated genes was taken as their expression. We then combined the two datasets by limma and removed the batch effect, and analyzed the data distribution before and after combining the two datasets by principal component analysis (PCA). The anosim function in the vegan package of the R language was used to calculate the P and R values. For the microarray data, we used Perl scripts to preprocess the raw matrices of GSE104948 and GSE104954. Probe identification numbers were converted to gene symbols and empty probes were removed based on the annotation information contained in each platform file. When multiple probes were matched to the same gene, the average expression value was used to determine the expression level of the gene. If there is a large variation in the expression of a gene, log (Exp+1) is used to measure the expression level.

### 2.3 WGCNA and GSEA

The “WGCNA” R package (Langfelder and Horvath, 2008) was used to identify the key modules most closely related to AAV in the matrix of merged GSE129752 and GSE158163 expression profiles. We extracted the AAV group’s pediatric vasculitis activity score (PVAS) scores from the original dataset as input data for WGCNA. A sample clustering dendrogram was constructed using the cluster function, and the topological overlap matrix (TOM) was constructed using the pickSoftThreshold function by WGCNA to determine the optimal soft threshold. We used candidate power values (1–20) to determine the average connectivity and independence of various modules. Dynamic shear trees were used to identify gene modules. Next, we measured the association between modules and sample traits using gene significance (GS) values and module membership (MM) values, and key modules were identified. We set |GS| to >0.3 and |MM| to >0.7 to filter hub genes in accordance with the official WGCNA guidelines and prior application examples so as to obtain the genes most relevant to the traits in the key module (Langfelder and Horvath, 2008; Tang et al., 2018). We performed GSEA (Wu et al., 2021b) using the KEGG and REACTOME gene sets in the GSEA dataset [c2.cp.v7.2.symbols.gmt (curated)]. A false discovery rate of <0.25 and *p*.adjust <0.05 (Subramanian et al., 2005) were set as the screening criteria for enrichment pathways.

### 2.4 Identification of co-key genes and GO/KEGG enrichment analysis

The module hub genes of WGCNA were intersected with the AAV-related key genes and nephropathy-related key genes in the Genecards database (Supplementary Table S1) to identify co-key genes of AAV. The “bioconductor” package “org.Hs.e.g.,.db” (Carlson, 2022) and “clusterProfiler” package were installed in R (R Foundation for Statistical Computing, Vienna, Austria), which was used for GO (Ashburner et al., 2000) analysis and KEGG (Kanehisa and Goto, 2000) pathway enrichment analysis while adopting *p* < 0.05 and *p*.adjust <0.05 as criteria to screen the co-key genes.

### 2.5 Identification of co-key genes associated with AAV-related renal injury

The expression levels of co-key genes in the glomerular dataset GSE104948 and the tubular dataset GSE104954 were identified. We extracted the gene expression information after preprocessing the data and performed the Wilcoxon rank sum test. Then, we used the “PRROC” (Gatto et al., 2014) package in R to examine the diagnostic efficacy of co-key genes by performing receiver operating characteristic (ROC) curve analysis.

### 2.6 NMF and ssGSEA

NMF (Gaujoux and Seoighe, 2010) clustering was performed on the eight co-key genes in the GSE104948 and GSE104954 datasets to identify potential features of interest in the gene-expression profiles. The distinct molecular subtypes of AAV-related renal injury were identified based on a cophenetic and consensus matrix. These distinct molecular subtypes were systematically assessed by ssGSEA analysis using the “GSVA” (Hänzelmann et al., 2013) R package to explore the immune infiltration and mechanisms of different subtypes of AAV-related renal injury.

### 2.7 Clinical significance of co-key genes

The Nephroseq database (http://v5.nephroseq.org/) (Zheng et al., 2017) is an online, free public platform. A correlation analysis of co-key genes and clinical features using the Nephroseq database was performed to evaluate the clinical prognostic value of key genes for AAV-related renal injury.

## 3 Results

### 3.1 WGCNA identified key module genes associated with AAV pathogenesis

**FIGURE 2:**
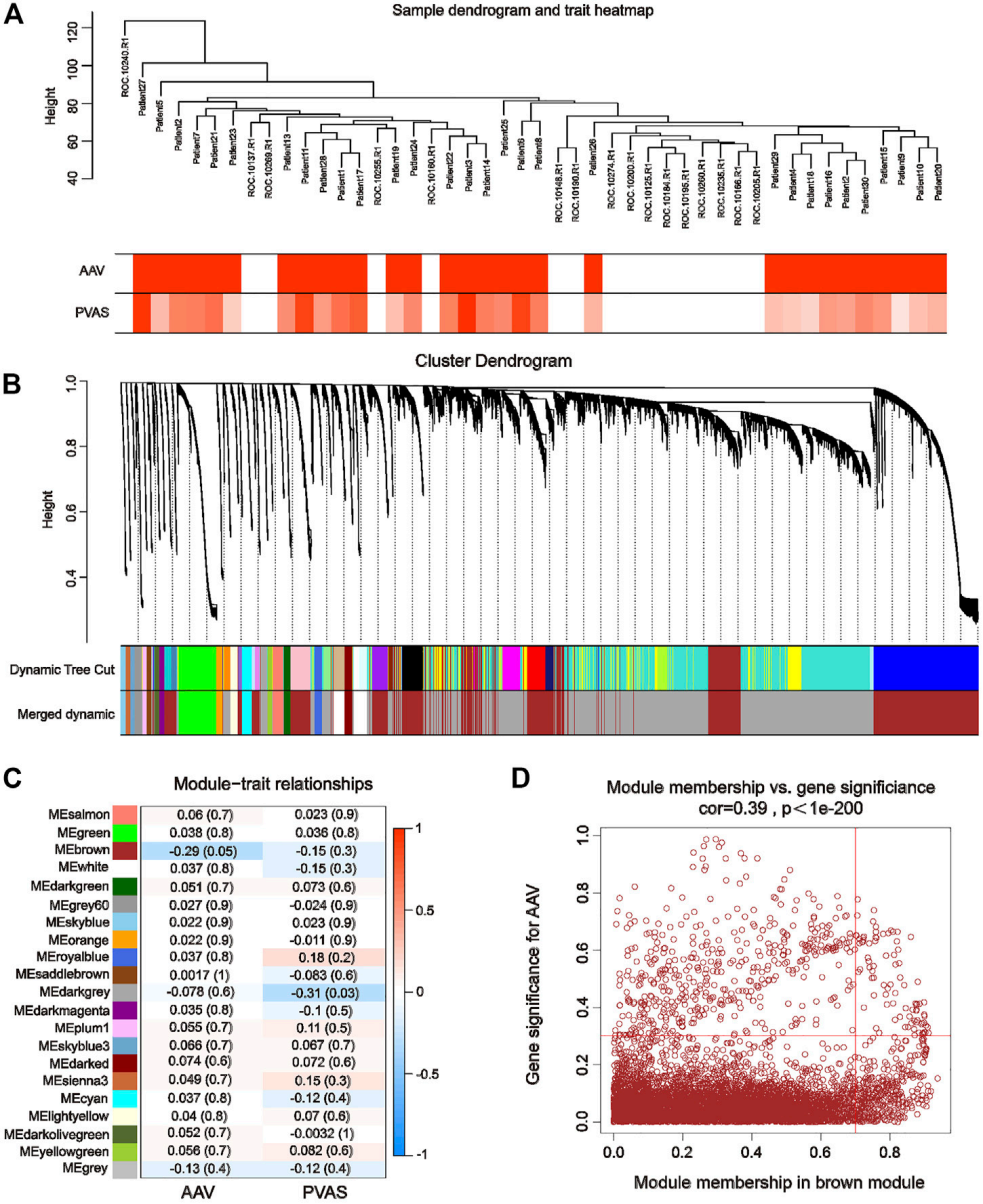
Weighted gene co-expression network analysis using GSE129752 and GSE111459. **(A)** Sample-trait clustering heatmap. **(B)** Dynamic shearing tree merging similar module genes. **(C)** Module-trait correlation heatmap. **(D)** Scatterplot for correlation between gene module membership in the brown module and gene significance.

The WGCNA package was performed to identify the key modules most closely related to AAV in the matrix of merged GSE129752 and GSE158163 expression profiles (which contained a total of 21,113 genes). Differences in the integrated dataset after removal of batch effects were mainly driven by intrinsic heterogeneity between the Disease and Control groups (Supplementary Figure S1). The control and AAV sets in WGCNA were automatically clustered into different branches (Figure 2A). The merged shear height of the dynamic shear tree was 0.85 for module identification. The minimum number of genes in each network module was set to 50, resulting in a total of 21 gene modules (Figure 2B). The most strongly correlated positive and negative modules were chosen as critical modules for discerning the pathogenesis of AAV traits. Our results indicate that the brown module (containing a total of 7,290 genes) was significantly negatively correlated with the AAV trait (cor = −0.29, *p* = 0.05) (Figure 2C). By setting |GS| > 0.3 and |MM| > 0.7 as criteria to screen the critical module genes for the pathogenesis of the AAV traits, we identified 462 genes associated with AAV pathogenesis (Figure 2D).

### 3.2 GSEA identified immune-related inflammation as a key molecular mechanism for AAV progression

**FIGURE 3:**
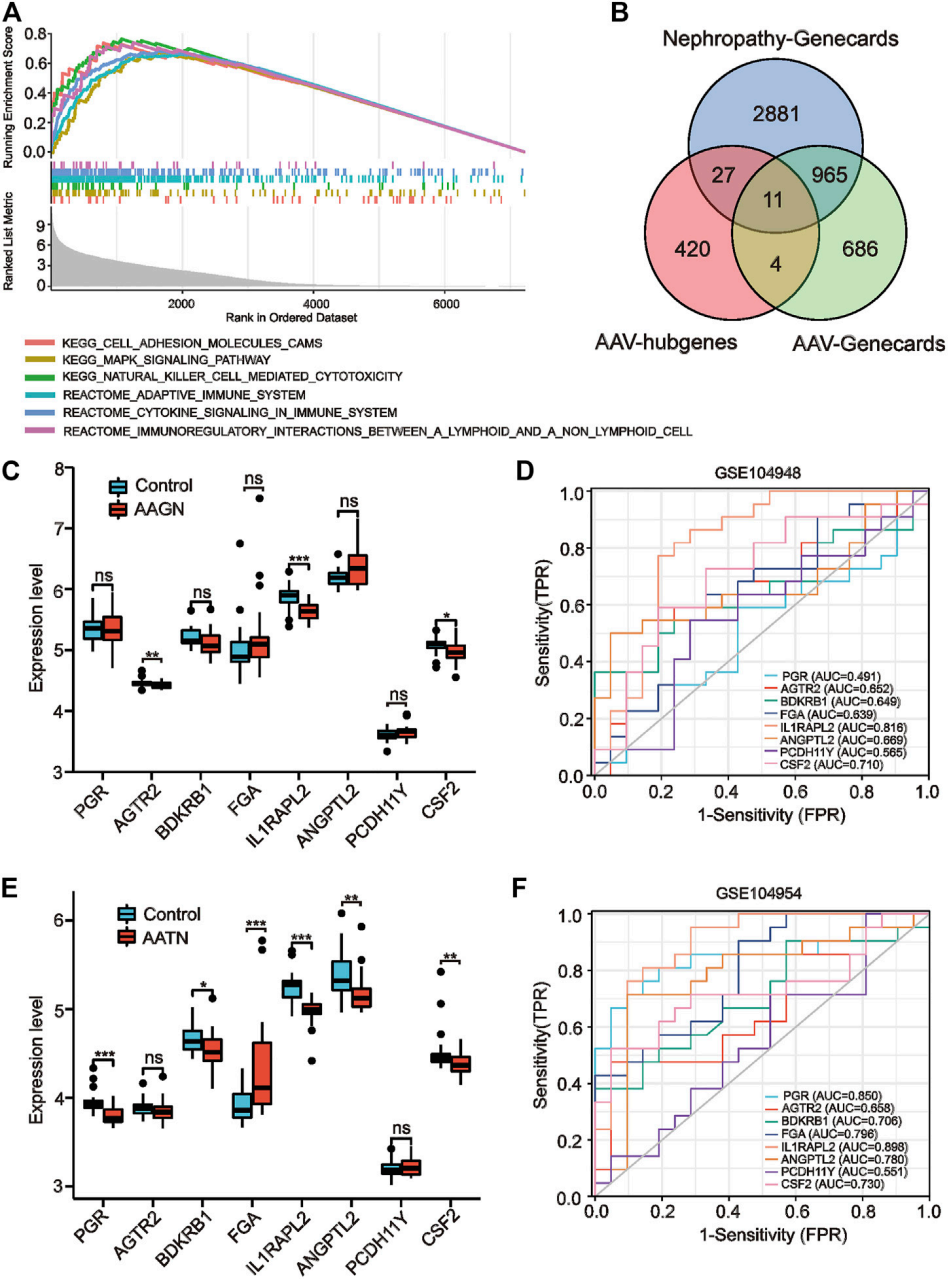
Gene set enrichment analysis (GSEA) enrichment analysis and validation of co–key genes in independent datasets. **(A)** GSEA enrichment results. **(B)** Venn diagram screening for co-key genes. **(C, E)** Expression of co-key genes in the GSE104948 and GSE104954 datasets for kidney injury in anti-neutrophil cytoplasmic antibody–associated vasculitis (AAV) (*p < 0.05, **p < 0.01, ***p < 0.001; ns, not statistically significant). **(D, F)** Receiver operating characteristic curves of eight key genes specific to kidney injury in AAV in GSE104948 and GSE104954.

We performed a comprehensive GSEA analysis of the screened brown module genes to discover the function and pathways of the key modules and explore the pathogenesis of AAV. The results showed (Figure 3A) that module genes were significantly enriched in the following immune- and inflammation-related pathways: antigen processing and presentation, autoimmune thyroid disease, immunomodulatory interactions, natural killer cell–mediated cytotoxicity, MAPK signaling pathways, and *Leishmania* protozoa infection. It has been suggested that immune, infection, and cellular responses are critical for the progression of AAV-related renal injury, which was consistent with the existing knowledge about AAV, and the accuracy of the module genes was also verified.

### 3.3 Identification of co-key genes associated with AAV-related renal injury

The WGCNA module hub genes were then intersected with the AAV-related key genes and nephropathy-related key genes in the Genecards database to identify key genes of AAV-related renal injury. We identified a total of 11 co-key genes, including *PGR*, *AGTR2*, *BDKRB1*, *FGA*, *1L1RAPL2*, *ANGPTL2*, *PCDH11Y*, *CSF2*, *ADAMTSL1*, *MIRLET7E*, and *SAA4* (Figure 3B); then, we validated the expression levels of these co-key genes in the ANCA glomerular dataset GSE104948 and the ANCA tubular dataset GSE104954 to verify that they have similar expression profiles in independent datasets. Since the expression profiles of *MIRLET7E*, *ADAMTSL1* and *SAA4* were missing in the independent dataset, we only validated the expression profiles of the remaining eight genes. Our results indicated that the expression levels of *PGR*, *AGTR2*, *BDKRB1*, *FGA*, *ANGPTL2*, and *PCDH11Y* in the GSE104948 expression profile data were not significantly different between the control and AAGN groups (Figure 3B). However, the expression levels of *1L1RAPL2* (*p* < 0.001) and *CSF2* (*p* < 0.05) were significantly lower in the AAGN group than in the control group (Figure 3C).

In the tubular dataset GSE104954, the expression levels of *PGR, BDKRB1, FGA, 1L1RAPL2, ANGPTL2,* and *CSF2* were significantly downregulated in patients with AATN (Figure 3E). We examined the diagnostic sensitivity and specificity of the eight hub genes by performing ROC curve analysis. In the glomerular dataset GSE104948, the area under the ROC curve (AUC) values for *1L1RAPL2* and *CSF2* were both >0.7, suggesting that these genes have good diagnostic efficacy as AAGN markers, with the larger AUC value being 0.816 (*1L1RAPL2*) (Figure 3D). In the tubular dataset GSE104954, as shown in Figure 3F, the AUC values of all six genes were >0.7 (*PGR*: 0.850, *BDKRB1*: 0.706, *FGA*: 0.796, *1L1RAPL2*: 0.808, *ANGPTL2*: 0.780, and *CSF2*: 0.730), suggesting that these six genes could be AATN biomarkers with high specificity. These genes have good diagnostic sensitivity and specificity for AAV-related renal injury.

### 3.4 Functions and signaling pathways of key genes involved in AAV-related renal injury

**FIGURE 4:**
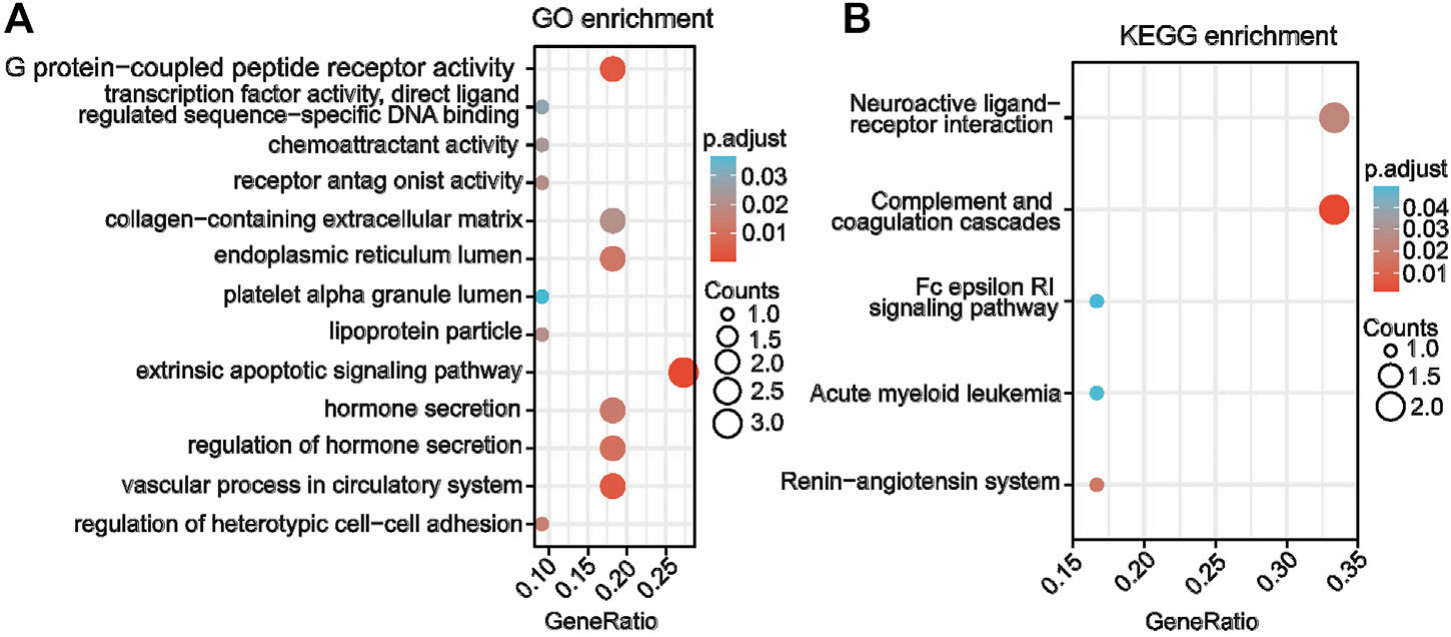
Gene enrichment analysis of co–key genes for AAV related renal injury **(A)** Gene Ontology enrichment analysis of co-key genes. **(B)** Kyoto Encyclopedia of Genes and Genomes enrichment analysis of co-key genes.

The biological functions and key signaling pathways of the eight candidate key genes were analyzed using GO and KEGG enrichment analyses. Our results showed that the co-key genes were mainly enriched for GO entries, including G protein–coupled peptide receptor activity, transcription factor activity, chemoattractant activity, receptor antagonist activity, endoplasmic reticulum lumen, platelet alpha granule lumen, extrinsic apoptotic signaling pathway, hormone secretion, vascular processes in the circulatory system, heterotypic cell–cell adhesion regulation, and other processes (Figure 4A). The KEGG pathways involved those designated for complement and coagulation cascades, the renin–angiotensin system, neuroactive ligand–receptor interaction, acute myeloid leukemia, the Fc epsilon RI signaling pathway, and others (Figure 4B).

### 3.5 Identification of new subtypes and immune infiltration based on NMF clustering

**FIGURE 5:**
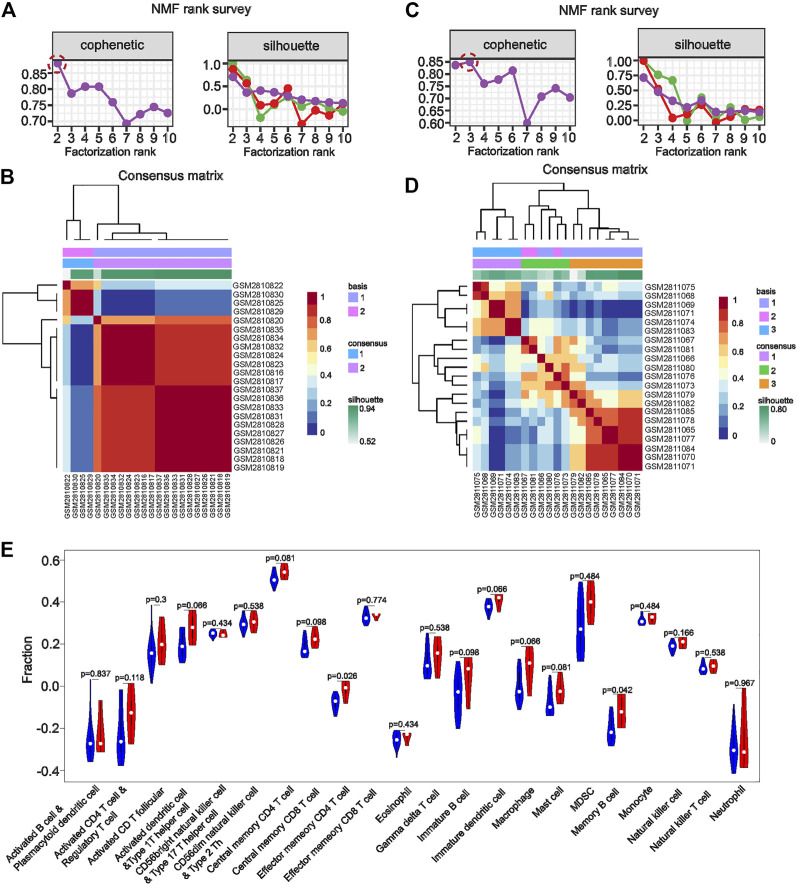
Non-negative matrix factorization clustering with eight co–key genes or molecular subtype identification in the glomerular dataset GSE104948 and the tubular dataset GSE104954. **(A, C)** The sharp decrease in the cophenetic correlation coefficient at rank r = 2 indicates that co-key genes could classify glomerular disease in anti-neutrophil cytoplasmic antibody–associated vasculitis (AAV)-related kidney injury into two subgroups. **(B, D)** In the tubular dataset GSE104954, the highest cophenetic correlation coefficient at rank r = 3 suggested that the co-key genes could classify renal tubular disease in AAV-related kidney injury into three subgroups. **(E)**. Immune cell infiltration of AAV-glomerular injury molecular subtypes. Immune infiltration analysis of molecular subtypes endows subtypes with immunological significance to the disease. High expression of both subtypes in a particular immune cell may suggest that the immune cell was essential to disease development The statistical differences obtained by comparing one subtypes with another subtype imply that a certain subtype plays a major role in that single immune cell.

To further explore whether the identified co-key genes are meaningful across renal injury subtypes, we performed NMF clustering with the eight co-key genes for molecular subtype identification using the glomerular dataset GSE104948 and the tubular dataset GSE104954. In the glomerular dataset GSE104948, a sharp decrease in the cophenetic correlation coefficient at rank r = 2 indicated that the co-key genes could classify glomerular disease in AAV-related kidney injury into a pair of subgroups (Figures 5A, C). Meanwhile, in the tubular dataset GSE104954, the highest cophenetic correlation coefficient at rank r = 3 suggested that the co-key genes could classify renal tubular disease in AAV-related kidney injury into three subgroups (Figures 5B, D). Importantly, these findings may offer a novel approach to the classification of AAV-related kidney injury. Next, we further investigated the immune infiltration among these five subtypes. The results of ssGSEA showed that, between both subtypes of AAGN, subtype 1 had a higher level of infiltration by effector memory CD4 T-cells and memory B-cells than subtype 2, suggesting that each cell type has a crucial role in the progression of subtype 1 AAGN renal injury (Figure 5E). Separately, the distribution of the immune infiltration of 28 immune cells in the three subtypes of AATN is shown in Supplementary Figure S2; notably, CD56dim natural killer infiltration in subtype 1 of AATN was significant (*p* = 0.048), and neutrophil cell infiltration in subtype 2 was significant (*p* = 0.0097), while memory B-cell infiltration was significant in subtype 3 (*p* = 0.0024). This indicated that these three cell types have a significant effect on the progression of the three subtypes of AATN.

### 3.6 Correlation analysis of key genes of AAV-related renal injury associated with clinical traits of kidney disease

**FIGURE 6:**
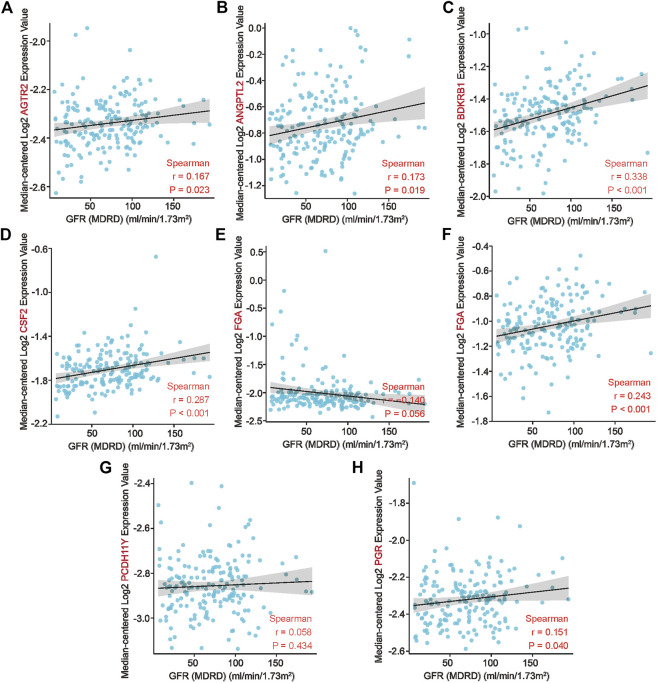
Association of co–key genes and clinical features in the Nephroseq database. **(A–H)** Correlation of eight co-key genes with the glomerular filtration rates of patients.

In summary, we determined that *AGTR2, ANGPTL2, BDKRB1, CSF2, FGA, IL1RAPL2, PCDH11Y,* and *PGR* are key genes in patients with AAV-related kidney injury, with diagnostic sensitivity and specificity. We further validated the relationship between co-key genes and clinical traits in the Nephroseq database to assess the association between co-key genes and prognostic factors of AAV-related kidney injury. Low expression of co-key genes was associated with a low glomerular filtration rate (*BDKRB1*: r = 0.338, *p* < 0.001; *PCDH11Y*: r = 0.287, *p* < 0.001; *IL1RAPL2*: r = 0.243, *p* < 0.001) (Figures 6A–H), suggesting that the expression of these genes may participate in the process of AAV-related kidney injury.But it is not clear whether its correlation can predict its poor prognosis, which requires further experiments and large clinical cohorts for validation.

## 4 Discussion

We identified 11 key genes of AAV-related renal injury. Eight of these genes were validated in independent datasets. These eight genes have good diagnostic efficacy suggests that they can serve as novel biomarkers and provide a new way to classify AAV-related renal injury. In addition, a comprehensive functional and pathway enrichment approach revealed that the biological mechanisms mediating the development of AAV-related renal injury are interrelated. Currently, there is a lack of effective, non-invasive biomarkers for AAV-related kidney injury; therefore, further exploration of the underlying mechanisms of the biological processes responsible for AAV-related kidney injury would contribute to the early diagnosis and treatment of the disease and improve the prognosis. The co-expressed genes identified in this study by integrating multiple approaches may be critical for elucidating the pathogenesis of AAV-related kidney injury.

A total of 462 key genes of AAV-related renal injury were screened using WGCNA. Multi-dataset integration analysis identified and validated eight co-key genes. GSEA showed that the key genes were significantly enriched in immune- and inflammation-related pathways. The key genes identified herein were those mainly enriched for GO entries, including G protein–coupled peptide receptor activity, transcription factor activity, chemoattractant activity, and other processes. The KEGG pathways of interest included those indicated for complement and coagulation cascades, the renin–angiotensin system, and others. The complement system plays a vital role in the development of AAV (Jennette and Falk, 2014a; Chen et al., 2017). Through bioinformatics techniques, we found that *AGTR2, ANGPTL2, BDKRB1, CSF2, FGA, IL1RAPL2, PCDH11Y*, and *PGR* are differently expressed in AAV patients and healthy people. In the validation of the independent microarray dataset, our results showed that 1L1RAPL2 and CSF2 expressed significantly lower levels in the GSE104948 expression profile data than the control group. In the tubular dataset GSE104954, *PGR*, *BDKRB1*, *FGA*, *1L1RAPL2*, *ANGPTL2*, and *CSF2* were significantly downregulated in AATN patients. Suggesting that the six genes have distinct differences in the renal tubules. Similarly, in the ROC curve analysis, the AUC values of all six genes were >0.7 in the renal tubular dataset, suggesting that these six genes could be AATN biomarkers with high specificity. These genes have good diagnostic sensitivity and specificity for AAV-related renal injury.The mechanism may be related to the following gene functions, but requires robust experiment to validate.

Among the eight key genes identified for AAV kidney injury, *CSF2* is involved in the pathogenesis of several inflammatory and autoimmune diseases, and play an essential role in regulating the pro-inflammatory function of monocytes in the pathogenesis of AAV-related renal injury (Krebs et al., 2017; VVilar et al., 2020; Rousselle et al., 2022). Rousselle (Jing et al., 2022) found that *CSF2* increased the ability of ANCA-stimulated monocytes to produce interleukin (IL)-1β and promote Th17 effector cell polarization. FGA is a major plasma protein coagulation factor in the regulation of wound healing, tissue regeneration, and the inflammatory response. *ANGPTL2* is an influential member of the angiopoietin-like protein in angiogenesis, lipid metabolism, and the maintenance of tissue homeostasis. Fang et al. (Fang et al., 2020) observed that irbesartan protects the kidney by inhibiting *ANGPTL2* and protecting the podocytes. *AGTR2* is a type of angiotensin II receptor whose activation leads to nitric oxide release and subsequent hyperpolarization, exerting anti-inflammatory and anti-fibrotic responses and inhibiting proliferation (Ghadhanfar et al., 2017). Activation of *BDKRB1*, a subtype of the bradykinin receptor, is induced by injury, pain, and intense inflammatory responses (Hofman et al., 2016; Girolami et al., 2021), making it a possible therapeutic target in chronic inflammation. Kakoki et al. (Kakoki et al., 2007) showed that *BDKRB1* plays an essential role in DNA damage, apoptosis, morphological and functional changes in the kidneys, and mortality during renal ischemia/reperfusion injury. *IL1RAPL2* is a molecule in the IL1R family with different biological effects on immune and inflammatory responses (Boraschi and Tagliabue, 2013). There is a lack of studies about *IL1RAPL2* and *PGR* in relation to kidney disease. Although their molecular mechanisms are not completely understood, we believe that this may be the first time that they have been identified as specific biomarkers of AAV-related renal injury, and further exploration of their effects on the mechanism of AAV-related renal injury is warranted.

We performed NMF clustering with the eight co-key genes for molecular subtype identification using the glomerular dataset GSE104948 and the tubular dataset GSE104954. In the glomerular dataset GSE104948, the co-key genes could classify in AAV-related kidney glomerular injury into two distinct subgroups. Meanwhile, in the tubular dataset GSE104954, the co-key genes could classify renal tubular disease in AAV-related kidney injury into three subgroups. Immune infiltration analysis of molecular subtypes endows subtypes with immunological significance to the disease, and immune cells associated with subtypes may potentially be significant to the disease. The statistical differences obtained by comparing subtypes with each other imply that a certain subtype plays a major role in a single immune cell. Analyzing the immune infiltration of different subtypes showed that effector memory CD4 T-cells and memory B-cells have an essential role in the progression of AAGN subtype 1. Meanwhile, CD56dim natural killer cells, neutrophils, and memory B-cells had a significant effect on the progression of the three subtypes of AATN. CD4^+^ effector T-cells mediate the production of neutrophil chemotactic factors through the release of IL-17A. Velden et al. detected numerous polymorphonuclear neutrophils expressing IL-17 in the glomerular and tubular mesenchyme. During the acute phase of the disease, neutrophils are an essential early intrinsic source of IL-17, helping to mediate ongoing renal inflammatory injury (Nogueira et al., 2010; Velden et al., 2012). B-cells can present antigens to T-cells, stimulate T-cell activation, and secrete pro-inflammatory factors (such as IL-6 and tumor necrosis factor) (McClure et al., 2018; Herrnstadt and Steinmetz, 2021). These results suggest that immune cells play an influential role in the progression of AAV-related renal injury (Lee, 2018; Soukou et al., 2021). The expression of these key genes positively correlates with the glomerular filtration rate, and their expression levels may be instructive for the prognosis of AAV-related renal injury.

In summary, this study systematically explored the potential mechanisms of AAV-related renal injury through comprehensive bioinformatics analysis and screened a number of biological genes and pathways that may be useful in the search for new biomarkers and therapeutic targets in AAV-related renal injury.

## 5 Conclusion

We found that *AGTR2*, *ANGPTL2*, *BDKRB1*, *CSF2*, *FGA*, *IL1RAPL2*, *PCDH11Y*, and *PGR* are potential novel biomarkers of AAV-related renal injury. These genes may be involved in the progression of AAV-related kidney injury through immune- and inflammation-related pathways. The co-key genes could be used to classify AAV-related kidney injury into different injury subtypes with differential patterns of immune infiltration that may closely relate to the pathogenesis of AAV-related kidney injury. Further exploration of the co-key genes’ effects on the molecular mechanism of AAV-related renal injury is warranted.

## Data Availability

The original contributions presented in the study are included in the article/Supplementary Material, further inquiries can be directed to the corresponding authors.

## Supplementary material

SUPPLEMENTARY TABLE S1 List of key AAV- and nephropathy-related genes in the Genecards database.
