# Supplementary material for: Identification of new immune subtypes of renal injury associated with anti-neutrophil cytoplasmic antibody–associated vasculitis based on integrated bioinformatics analysis
Source: Front Genet. 2023 Apr 5;14:1119017. doi: 10.3389/fgene.2023.1119017 (PMC10113532; doi:10.3389/fgene.2023.1119017)
Supplement: Supplementary file 2 [file DataSheet1.docx]

Supplementary Material

**Identification of new immune subtypes of renal injury associated with anti-neutrophil cytoplasmic antibody–associated vasculitis based on integrated bioinformatics analysis**

**Lizhen Lin^#^, Keng Ye^#^, Fengbin Chen, Jingzhi Xie, Zhimin Chen*, Yanfang Xu***

*** Correspondence:**

Corresponding Author:

Zhimin Chen: chenzm0905@163.com (Z.C)

Yanfang Xu: xuyanfang99@hotmail.com (Y.X.)

## Supplementary Figures


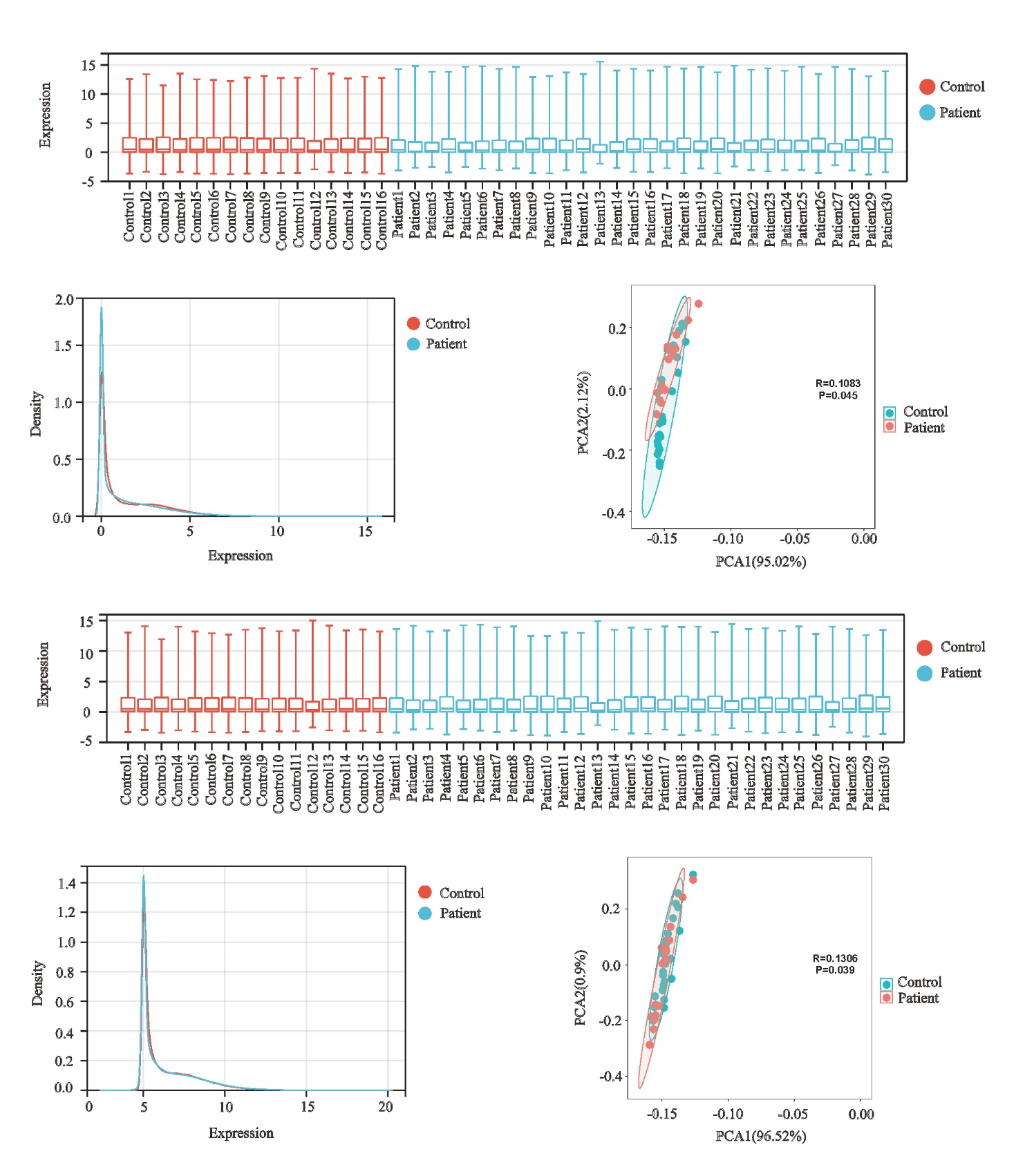


**Supplementary Figure S1 |** Data for WGCNA before and after removal of batch effects.(A) Control and Patient were obtained from two different datasets, and the distribution of the data for each sample before removing the batch effect. (B) Distribution density of each sample data before removal of batch effect. (C) The principal component analysis of the two sets of data before removing the batch effect, and the vegan package of R language was used for P-value and R-value calculation.(D) The distribution of the data for each sample after removing the batch effect. (E) Distribution density of each sample data after removal of batch effect. (F) The principal component analysis of the two sets of data after removing the batch effect. After removing batch effects, the differences between the two data sets were statistically significant, with differences mainly arising from the inherent heterogeneity of disease and control.


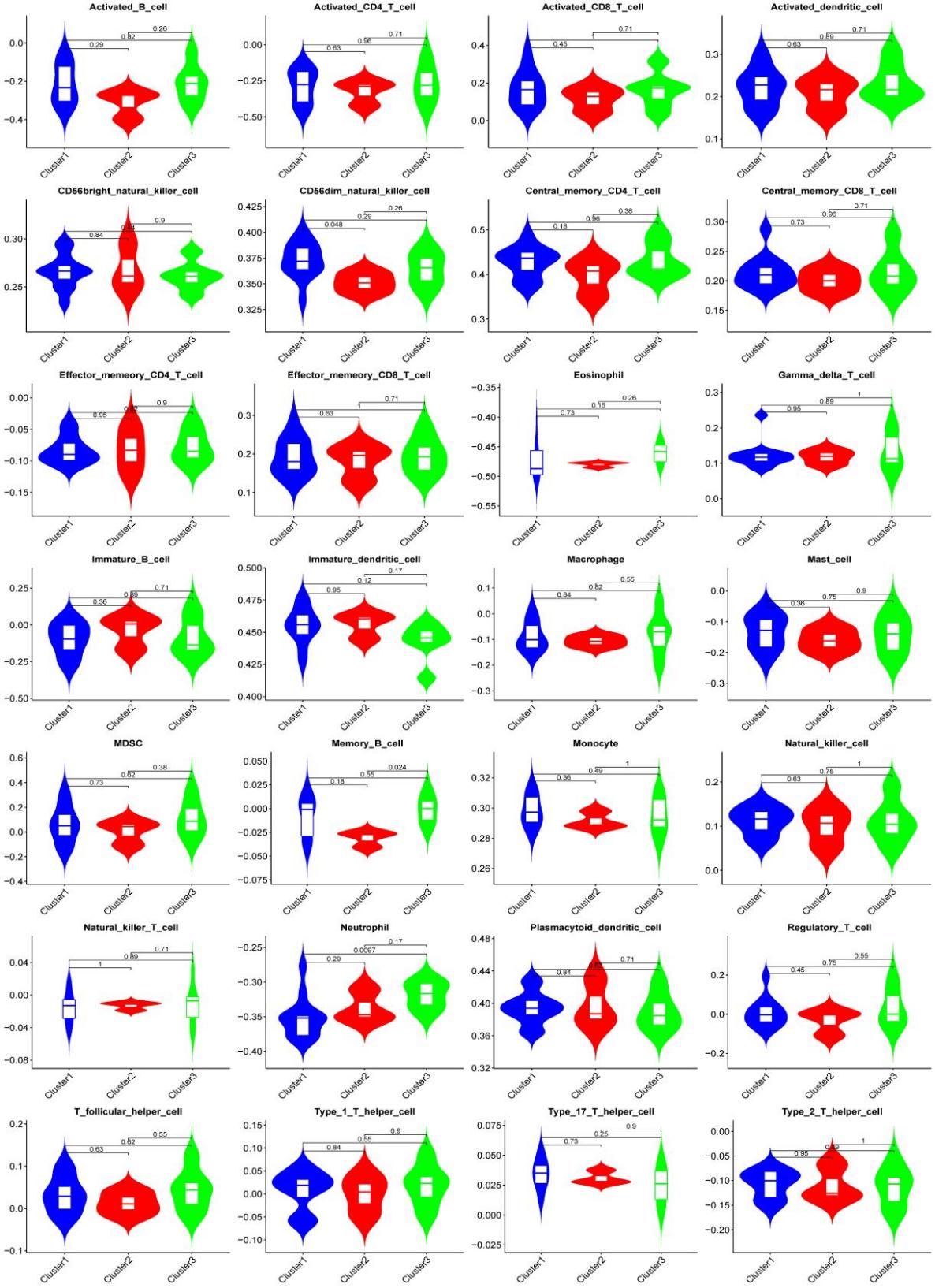


**Supplementary Figure S2.** **Analysis of the immune landscape associated with anti-neutrophil cytoplasmic antibody–associated vasculitis (AAV)-related renal injury.** The distribution of 28 immune cells in three different subtypes in the tubular dataset GSE104954 of AAV-related renal injury is shown. Immune infiltration analysis of molecular subtypes endows subtypes with immunological significance to the disease. High expression of both subtypes in a particular immune cell may suggest that the immune cell was essential to disease development The statistical differences obtained by comparing one subtypes with another subtype imply that a certain subtype plays a major role in that single immune cell.
